# Mineralization of Trifluoromethanesulfonate via Subcritical Hydrothermal Reaction: Reaction Mechanism and Transformation Pathways

**DOI:** 10.1021/acs.est.6c00018

**Published:** 2026-06-25

**Authors:** Shilai Hao, Benjamin Payton, Ori Soker, Anderson Ellis, Sean Brooks, Patrick Reardon, Shubham Vyas, Ilja Popovs, Igor Novosselov, Christopher P. Higgins, Timothy J. Strathmann

**Affiliations:** † Department of Civil and Environmental Engineering, 3557 Colorado School of Mines, Golden, Colorado 80401, United States; ‡ School of Plant and Environmental Sciences, Virginia Tech, Blacksburg, Virginia 24061, United States; § Department of Chemistry, 3557 Colorado School of Mines, Golden, Colorado 80401, United States; ∥ NMR Facility, Oregon State University, Corvallis, Oregon 97331, United States; ⊥ Department of Nuclear Engineering, 122608 University of Tennessee, Knoxville, Tennessee 37996, United States; # Department of Mechanical Engineering, University of Washington, Seattle, Washington 98195, United States

**Keywords:** ultrashort-chain PFAS, nucleophilic substitution, nuclear magnetic resonance (NMR), formate, hydrogen

## Abstract

Hydrothermal alkaline treatment (HALT) is an innovative approach that was developed for the destruction of per- and polyfluoroalkyl substances (PFAS). While HALT has been shown to effectively destroy a wide range of PFAS detected in various sample matrices, a comprehensive understanding of the controlling reaction mechanisms and transformation pathways remains limited. Herein, we selected trifluoromethanesulfonate (TFMS), the shortest-chain and likely one of the most recalcitrant PFAS reported to date, to probe degradation mechanisms and identify transformation products. The results indicate that HALT of TFMS proceeds via general nucleophilic substitution and base-promoted pathways, leading to 65.7% mineralization of the parent compound after a 180 min reaction of 0.1 M TFMS at 350 °C in 1 M NaOH. For the degraded TFMS, approximately 100% of fluorine and sulfur were converted to fluoride and sulfate, respectively, while carbon was distributed mainly as carbonate (95%) and formate (5%), along with the production of hydrogen. These findings are supported by both experimental and computational evidence. Hydroxide plays dual roles by initiating the reaction as the nucleophile and promoting subsequent steps by maintaining strongly basic conditions. The initial degradation step is rate-determining, with an estimated energy barrier of 27.8 kcal/mol. Similar mechanisms are proposed for reactions of longer-chain perfluoroalkyl sulfonic acids (PFSAs). Finally, the key factors governing PFSA reactivity across chain lengths from C1 to C8 were identified as reaction temperature, nucleophile type and concentration, and reaction time. This study addresses a critical knowledge gap in PFAS hydrothermal reactions and further establishes HALT as an effective technology for PFAS destruction and defluorination.

## Graphical abstract

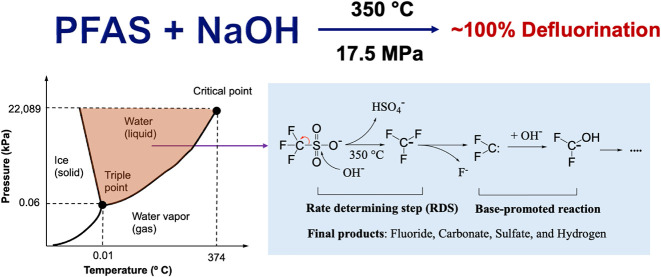

## Introduction

Per- and polyfluoroalkyl substances (PFAS) have become a global health concern due to their widespread occurrence,^1^ significant recalcitrance,^2^ bioaccumulation,^3^ and toxicity.^4^ To address the concern, the EU and the USA have issued strict PFAS regulations for drinking water and waste discharges to protect residents from PFAS exposure. For example, the U.S. EPA recently established strict drinking water Maximum Contaminant Level (MCL) regulations of 4 ng/L for perfluorooctanoic acid (PFOA) and perfluorooctane sulfonic acid (PFOS),^5^ while also designating the same compounds as hazardous substances under the Comprehensive Environmental Response, Compensation, and Liability Act.^6^ These regulations, in addition to state-level limits,^7^ have motivated intense efforts to develop effective and economical PFAS treatment technologies.

A number of innovative destructive approaches that target the breaking of the C–F bond are currently under development to end the cycle of PFAS contamination. These approaches, including photocatalytic,^8,9^ electrochemical,^10,11^ sonochemical,^12–14^ mechanochemical,^15–17^ plasma,^18,19^ hydrated electron,^20–22^ radiolytic,^23^ and subcritical and supercritical hydrothermal treatment processes,^24–32^ have shown promise for degradation of a broad range of PFAS in both dilute water matrices (e.g., drinking water)^21,24^ and concentrated wastes [e.g., aqueous film forming foam (AFFF) stockpiles].^25,33^ While published studies report varying degrees of degradation of various PFAS, most treatment technologies do not achieve complete defluorination (mineralization) of individual PFAS or PFAS mixtures in environmental/concentrated samples, leading to products of incomplete destruction, including short-chain perfluoroalkyl acids (PFAAs) and/or other partially fluorinated byproducts.^34–36^ In addition, several treatment technologies that are effective for degrading perfluoroalkyl carboxylic acids (PFCAs) are ineffective or significantly less effective for more recalcitrant perfluoroalkyl sulfonic acids (PFSAs).^22,37,38^ While investigators continue to make efforts to optimize each technology to enhance the destruction and reduce treatment costs, establishing the underlying defluorination mechanisms is essential for process design and optimization. A fundamental understanding of the reaction mechanism not only reveals the potential for the formation of toxic byproducts, but may also provide guidance on eliminating or minimizing their formation during treatment operations.

Subcritical hydrothermal alkaline treatment (HALT) is a thermochemical process that applies strong alkali in near-critical liquid water (e.g., 350 °C, ≥1 M NaOH, 17.5 MPa)^25,27^ to achieve near-complete destruction of a wide range of PFAS (≥145 PFAS examined to date) across various sample matrices, including AFFF stocks, AFFF-impacted soil and groundwater, spent granular activated carbon, foam fractionate, and synthetic water as a representative of industrial wastewater streams.^24–30,39^ Although previous studies have demonstrated HALT’s ability to achieve near-complete defluorination and have provided initial insights into its reaction mechanisms,^25,27,39^ stable fluorinated intermediates have not been observed during the treatment of real-world PFAS waste matrices. As a result, the underlying reaction mechanisms remain incompletely understood. In addition, previous studies have explored reaction kinetics models for PFAS mixtures;^25,27^ however, a systematic evaluation of the factors governing the degradation kinetics of ultrashort-chain PFSAs, such as trifluoromethanesulfonic acid (TFMS) and pentafluoroethanesulfonic acid (PFEtS), in comparison with short- and long-chain PFSAs, such as perfluorobutanesulfonic acid (PFBS) and PFOS, is still lacking. Addressing these knowledge gaps requires (1) identifying transformation intermediates and final products involving all constituent elements of TFMS (carbon, fluorine, oxygen, and sulfur), (2) elucidating the principles governing defluorination, and (3) investigating the factors that affect the degradation of ultrashort-chain PFSAs.

Herein, we selected TFMS, the shortest-chain and likely one of the most recalcitrant PFAS reported to date,^19,40^ as a model compound to probe the degradation mechanisms. The growing evidence of environmental occurrence and potential toxicity of ultrashort-chain PFAS,^41,42^ including TFMS, combined with their extreme recalcitrance toward treatment technologies,^43,44^ makes TFMS an ideal candidate for mechanistic investigation because of both its structural simplicity and environmental relevance. Transformation products of TFMS were identified via a comprehensive suite of analytical tools, including nuclear magnetic resonance (NMR) spectroscopy, gas chromatography with thermal conductivity detection (GC-TCD), and ion chromatography (IC). Tentative stepwise transformation pathways are proposed and supported by multiple lines of experimental and computational evidence. Building on the TFMS degradation mechanism, prior literature, and additional computational evidence, we also propose degradation mechanisms for longer-chain PFSAs. These proposed pathways are supported by kinetic data for PFSAs with chain lengths ranging from C1 to C8, which show comparable responses to key reaction conditions. This study fills a critical knowledge gap in the application of HALT for PFAS destruction and further validates its effectiveness as a PFAS remediation strategy that achieves complete mineralization without formation of stable partially fluorinated byproducts.

## Materials and Methods

### Chemicals and Materials

All PFAS, including PFSAs, PFCAs, 1H-perfluorooctane, perfluoro-1-heptene, and perfluorooctane, were purchased from Sigma-Aldrich (St. Louis, MO), Combi-Blocks (San Diego, CA), VWR, and Synquest Laboratories Inc (Alachua, FL). All PFAS analytical standards, including isotope-labeled internal standards (IS), were purchased from Wellington Laboratories as described previously.^28^ Details of other chemical reagents are provided in Table S1 in the (Supporting Information SI).

### Hydrothermal Reactions

Hydrothermal reaction of PFSAs was conducted in stainless steel batch reactors from Parr Instrument Company (4740 series; 25 mL total volume with overpressure safety relief mechanism; Figure S1a) using procedures described previously.^25^ Single-PFSA or mixed-PFSA solutions were amended with NaOH stock solutions and then rapidly heated to 350 °C in a fluidized sand bath (Figure S1c). The reactor headspace was not purged with an inert gas, such as N_2_ or He, prior to the reaction, so air remained present in the headspace during the reaction. Reactions were quenched after the desired reaction time (15–180 min) by removing the reactor from the heat source and cooling with an air fan. After cooling to room temperature, the reactor contents were collected using a plastic pipette, transferred into 15 mL conical polypropylene centrifuge tubes, and stored at 4 °C prior to analysis. All materials and containers had been previously tested and confirmed to be PFAS-free. All reactions were conducted in duplicate, with uncertainties reported as min/max observed for the replicates. Headspace samples were collected using a stainless-steel gas sampling valve (Swagelok, OH) and transferred into a 500 mL aluminum foil gas sampling bag (Restek Corporation, PA) for subsequent gas chromatographic analysis (Figure S1b).

To gain mechanistic insights into an ultrashort-chain PFSA, a concentrated TFMS stock solution (0.1 M; 14.9 g/L in water) was subjected to HALT to generate sufficient quantities of transformation intermediates and final products for detection by analytical instruments, including nuclear magnetic resonance (NMR) spectroscopy and IC.

To provide confirmation that the proposed mechanism extends to longer-chain PFSAs, HALT experiments were also conducted with PFBS (C4 PFSA) as well as potential intermediates associated with the first degradation step of long-chain PFSAs, including 1H-perfluorooctane, perfluoro-1-heptene, and perfluorooctane. As water solubilities of these potential intermediates are low (<1 mg/L) according to www.chemeo.com, pure standards were directly spiked into the reactor using a 100 μL syringe, and the reactor was sealed immediately after spiking. A relatively high initial concentration of approximately 1600 mg/L was used to ensure sufficient fluoride release could be detected if defluorination occurred.

Finally, a mixture of PFSAs spanning chain lengths from C1 to C8 was subjected to a range of HALT conditions to quantify their influence on reaction kinetics of the full suite of structures.

### Sample Analyses

PFAS analyses were performed by a UHPLC (Ultimate LPG-3400RS) coupled with an Orbitrap Exploris 240 high-resolution Orbitrap mass spectrometer (LC-Orbitrap-MS, Thermo Scientific). The fluoride ion (F^–^) concentration in post-treatment samples was measured with a fluoride ion-selective electrode (FISE). Prior work^25^ validated these measurements using IC. ^19^F- and ^13^C-NMR spectra were collected on either a JEOL ECA-500 spectrometer (500 MHz) or a Bruker Avance IIIHD 800 MHz spectrometer equipped with a 5 mm triple resonance cryogenic probe, with the latter offering higher sensitivity for low-concentration samples. Ion products from TFMS degradation, including formate and sulfate, were quantified by IC following a protocol outlined by Gao *et al*.^45^ Gas products were measured by GC-TCD. Post-treatment liquid samples were also analyzed to close carbon mass balance using a Shimadzu TOC-L analyzer operated in total carbon and inorganic carbon modes. Detailed descriptions of each analysis method are provided in the SI (Text S1-S4 and Figure S3).

### DFT Calculation

All quantum mechanical calculations were performed using the Gaussian 16 software suite.^46^ A Minnesota density functional, M06-2X, was utilized with triple zeta Pople basis set that included diffuse and polarization functions, 6–311+G­(d,p). The M06-2X functional was used due to its reputation for accurate predictions of thermochemical properties and kinetics of main group elements.^47^ All stationary points were identified as minimum energy structures or transition states by the presence of zero or one imaginary vibrational mode, respectively. In cases where there is no distinct transition state, such as cleavage of the carbon-fluorine bond of the trifluoromethyl carbanion leading to the fluoride ion and a difluorocarbene, the barrier and free energy of activation were obtained by relaxed potential energy surface scans using the minimum energy geometry of the reactant. All computed energies were corrected for temperature at 350 °C and pressures at 17.5 MPa to match HALT conditions. Corrections for temperature and pressure were made using the temperature and pressure keywords, which modify the corresponding parameters in the partition functions used to calculate thermodynamic quantities.^48^ A polarizable continuum model (PCM) with a dielectric constant of 13 was applied, as implemented in Gaussian 16, to approximate solvation under HALT conditions based on the dielectric behavior of subcritical water.^49^ The dielectric constant was selected from subcritical water properties from historical NIST data.^50^ To obtain accurate thermochemical data, hindered rotation corrections were also made. The necessity for hindered rotations arises from the treatment of rotational modes with a harmonic potential. With the presence of a perfluorinated alkyl chain, there is a perturbation on the typical rotational potential energy surface of a carbon–carbon bond.^51^ The activation barrier was obtained by subtracting the reactant or reactant complex free energies from the free energy of transition states, while the free energy of reaction was obtained by subtracting the sum of reactant free energies from the sum of product free energies.

## Results and Discussion

### Defluorination and Degradation Mechanism for TFMS

Experimental and computational studies were initiated with TFMS (CF_3_-SO_3_^–^), the C_1_ PFSA. While detailed investigation of each step in the reaction pathway and discussion of other possible mechanisms are presented in later sections, Figure 1 first illustrates the most likely overall reaction equation and proposed defluorination pathway of TFMS during HALT, supported by a series of combined experimental observations and computational simulations. Overall, TFMS was mineralized into fluoride, sulfate, formate, carbonate, and hydrogen through a combination of nucleophilic substitution and base-promoted hydrolysis reactions (Figure 1A).

**Figure 1:**
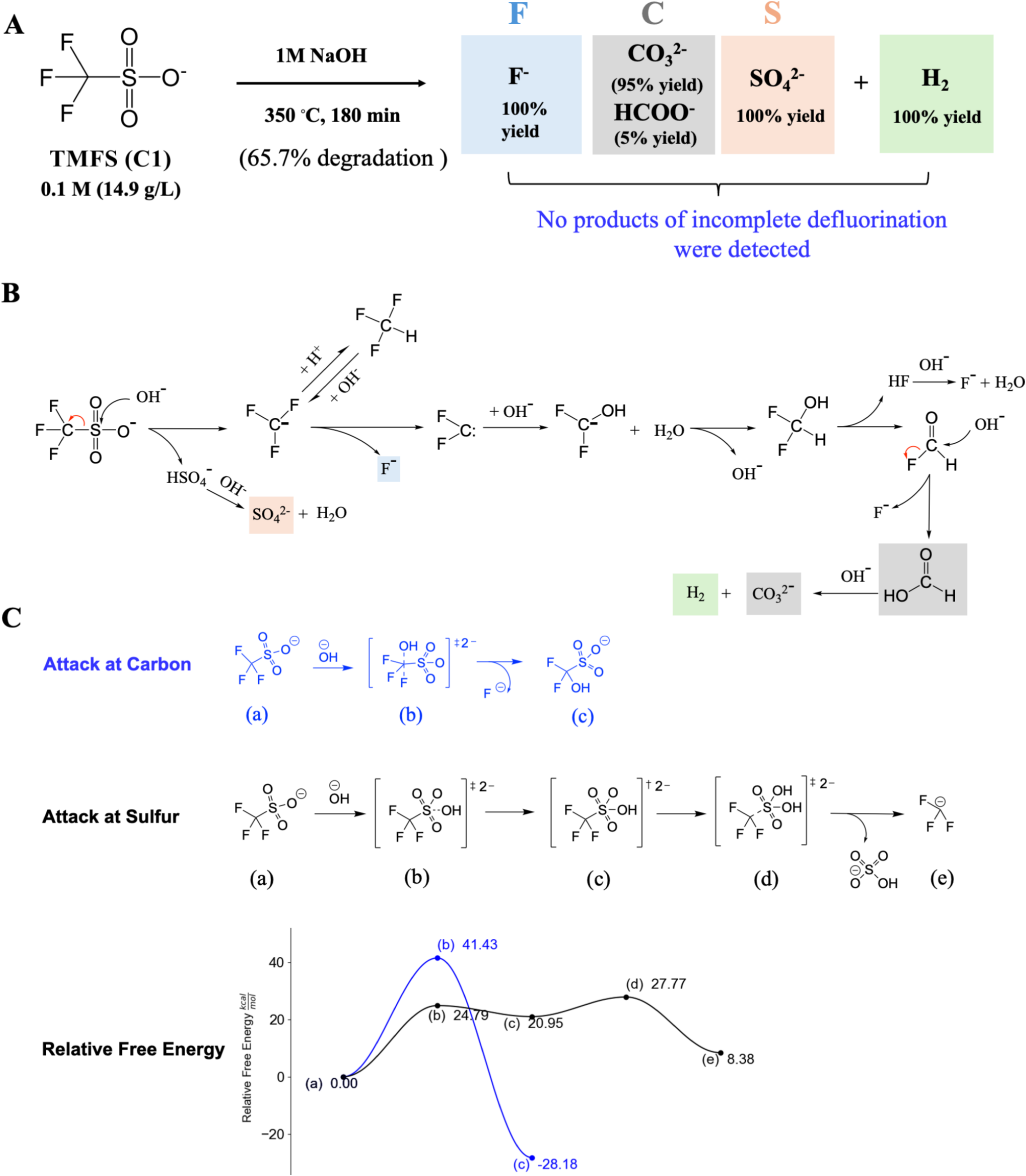
TFMS defluorination pathways during HALT. (A) Overall reaction stoichiometry and product yields for 0.1 M TFMS reaction at conditions: 350 °C, 1M NaOH, and 180 min. Highlighted structures represent products detected regarding each element in TFMS. Numbers in parentheses represent yields after 180 min of reaction. (B) Proposed mineralization pathway for TFMS. (C) Comparison of the energy barriers for OH^–^ attack on the carbon atom versus the sulfur atom in TFMS.

To obtain insights into the governing mechanism, a concentrated TFMS solution (0.1 M, 14.9 g/L in water) was subjected to HALT under conditions of 350 °C, 1M NaOH, and reaction times ranging from 15–180 min. First, the degradation, defluorination, and desulfonation (as indicated by sulfate yield) of TFMS occurred at almost equivalent rates throughout the reaction timecourse (Figure 2A). This suggests that C-S and C-F bond cleavages occur in rapid succession. DFT calculations, to be discussed below, indicate that an initial C-S bond cleavage is favored, followed by the rapid and complete defluorination of remaining CF_3_ moiety along with formation of sulfate (SO_4_^2–^). No stable or partially fluorinated intermediates (e.g., compounds with monofluoro or difluoro moieties) were observed in the aqueous phase sample throughout the reaction, a finding further supported by a time series of ^19^F-NMR spectra (Figure 2B). Decreases in NMR peaks associated with TFMS are accompanied by an increase in fluoride ion formation, with no additional fluorine signals detected as intermediates. Quantification of fluoride and sulfate confirmed that the fluorine and sulfur released from TFMS degradation were converted nearly simultaneously and completely into fluoride and sulfate, respectively. Notably, TFMS defluorination increased to 95 ± 1% when the reaction time was extended to 300 min, although approximately 40% of TFMS still remained after 180 min (Figure 1A). The very high initial TFMS concentration used in these mass balance tracking experiments (0.1 M, 14.9 g/L), which is close to the same order of magnitude as the initial NaOH concentration, reduced the extent of degradation observed compared to kinetics measurements reported in Figure 4 due to significant neutralization of OH^–^ as the reaction proceeds in the experiments using elevated initial TFMS concentration. In kinetics experiments conducted at a much lower initial TFMS concentration (0.014 mM) where OH^–^ neutralization would be minimal, near-complete degradation (99.6%) was observed with the same reaction time.

**Figure 2:**
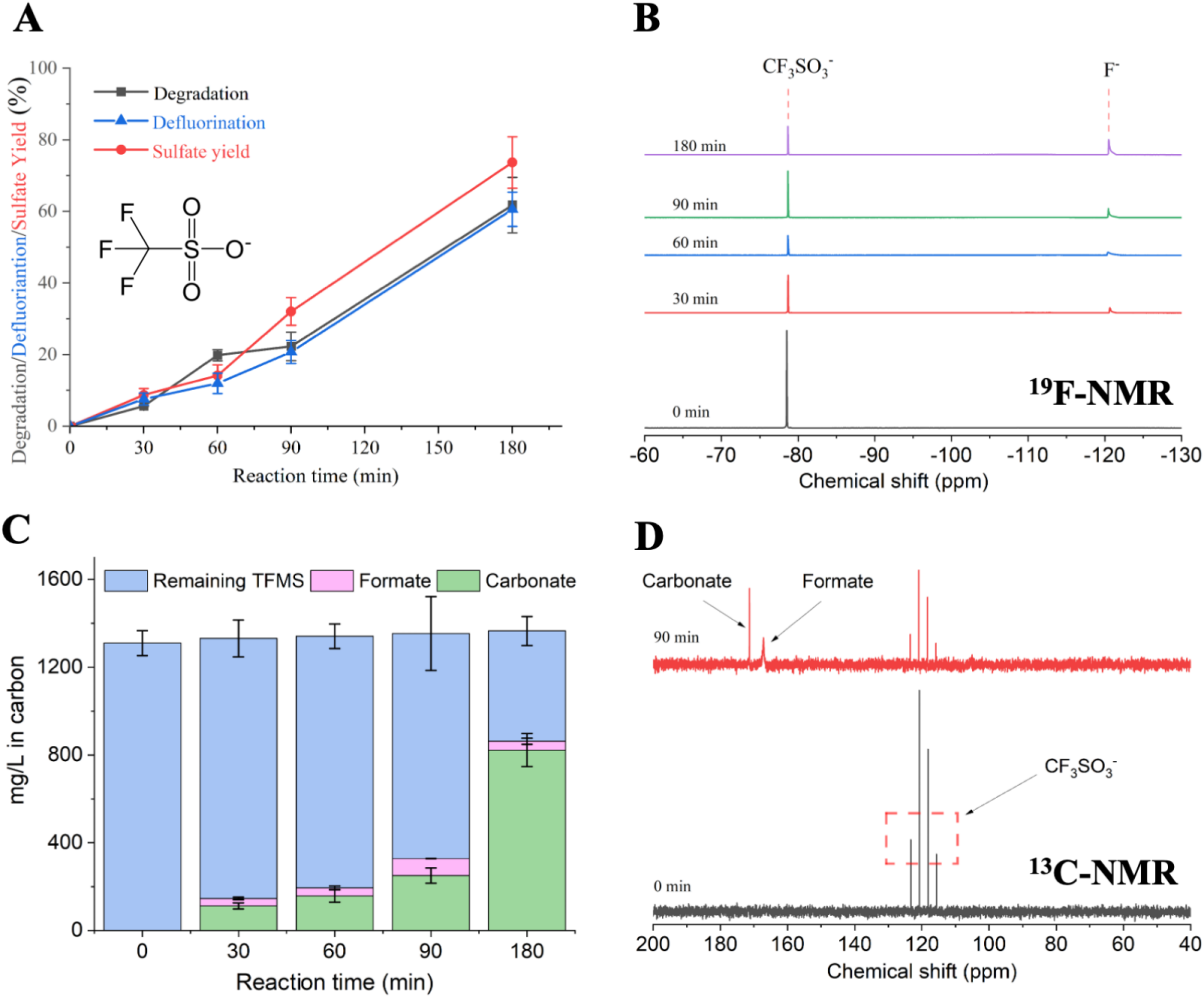
The monitoring of TFMS reaction during HALT at 350 °C with 1 M NaOH. (A) Degradation, defluorination, and sulfate formation as a function of reaction time; (B) ^19^F-NMR spectra; (C) carbon mass balance closure; (D) ^13^C-NMR spectra.

The fate of carbon within TFMS was tracked by ^13^C-NMR analysis of aqueous samples before and after HALT treatment (Figure 2D). Two new peaks (167.6 and 171.3 ppm), distinct from the carbon in TFMS, were identified as carbonate and formate anions after comparing with standards (Figure S2). The carbon mass balance was closed by quantifying the remaining TFMS, formate, and carbonate using IC and a TOC analyzer (Figure 2C). Headspace analysis of the reactor using GC-TCD revealed up to 30% v/v H_2_ in a total gas volume of 53 mL collected after the reaction (350 °C, 1 M NaOH, and 90 minutes), with the remainder consisting mainly of N_2_ derived from residual air trapped in the reactor headspace (Figure S4). Notably, more comprehensive evaluation of both fluorinated and non-fluorinated gaseous products should be considered in future work, particularly as analytical methods for volatile PFAS continue to develop, such as U.S. EPA Other Test Method 50 (OTM-50).^52^ The experimentally measured H_2_ production (15.9 mL) closely aligned with the theoretical stoichiometric yield from Figure 1A (14.7 mL; details of calculation provided in Figure S4). Based on the identified transformation products and mass balance closure for carbon, sulfur, and fluorine, we propose the stepwise mechanism shown in Figure 1B. This mechanism suggests that defluorination of TFMS occurs via a series of nucleophilic (OH^–^) substitutions and base-promoted hydrolysis reactions, consistent with previous reports on TFMS and chlorinated organics such as dichloromethane.^53,54^

As depicted in the proposed mechanism in Figure 1B, the OH^–^ nucleophile first attacks the sulfur atom leading to C-S bond cleavage in the rate-determining step, resulting in the production of CF_3_^–^ (carbanion) and HSO_4_^–^ (bisulfate).^47^ The bisulfate is subsequently converted to sulfate under basic conditions. The hydroxide attack at the sulfonate head group is suggested to be more favorable because the transition state is stabilized by the electron-withdrawing nature of the oxygen atoms and the fluorinated carbon. Another alternative scenario (Figure 1C) was also considered in which OH^–^ first attacks the carbon in the C-S bond, leading to the formation of CF_3_OH and SO_3_^2–^ (sulfite). This scenario was disfavored after considering the molecular orbitals of the carbon–sulfur bond. This can be explained by the higher-energy valence shell of sulfur, which suggests that a larger portion of the electron-deficient carbon–sulfur σ* orbital resides on the sulfur atom (Figure S5), making sulfur the preferred site for nucleophilic attack by the lone pair of electron-rich hydroxide.^55,56^ In addition, the surrounding fluorine atoms can sterically and electronically shield the carbon center, limiting the approach of hydroxide and making direct C–F bond cleavage/fluoride departure less favorable, which is also consistent with previous reports on organofluorine chemistry.^57,58^ Experiments, unfortunately, could neither confirm nor rule out either mechanism based solely on the sulfur species identification, as separate experiments show that SO_3_^2–^ is rapidly converted to SO_4_^2–^ under HALT reaction conditions (See Text S5). However, DFT calculations reveal a much lower energy barrier for OH^–^ nucleophilic attack on sulfur (27.8 kcal/mol) compared to carbon (41.4 kcal/mol) (see Figure 1C). The former also agrees closely with the apparent activation energy barrier determined from the experiments (26.8 kcal/mol). Collectively, these findings support an initial reaction step involving nucleophilic attack on the sulfur atom and C-S bond cleavage to form CF_3_^–^ and SO_4_^2–^.

CF_3_^–^ is also the initial fluoroalkyl intermediate proposed for alkali-independent hydrothermal decarboxylation of trifluoroacetic acid (TFA) by Austin et al.^59^ If formed in the absence of OH^–^, CF_3_^–^ abstracts a proton from water to form CF_3_H (trifluoromethane or fluoroform; Figure S7). In separate experiments, CF_3_H formation was observed during alkali-free reaction of TFMS at temperatures above 400 °C. However, under basic pH conditions required for HALT, CF_3_^–^ rapidly decomposes into fluoride and difluorocarbene (CF_2_:) (Figure 1B), as reported by Langlois *et al*.^53^ CF_3_^–^ is widely recognized as an unstable, transient intermediate unless specially stabilized, and it can readily undergo α-fluoride elimination to generate singlet difluorocarbene (CF_2_:) and fluoride.^60,61^ The instability of CF_3_^–^ has been attributed to unfavorable electrostatic interactions in the anion, whereas difluorocarbene is relatively stabilized by lone-pair donation from fluorine into the vacant p orbital of the carbene carbon.^60^ DFT calculations further indicate that formation of CF_2_: from CF_3_^–^ proceeds with an energy barrier of 17.9 kcal/mol. Although this barrier is non-trivial, it remains substantially lower than that of the initial nucleophilic substitution step (27.8 kcal/mol; Figure 1C). Once difluorocarbene (CF_2_:) is formed, it may either be trapped by an alkene to produce a gem-difluorocyclopropane or undergo self-reaction to form tetrafluoroethylene (CF_2_=CF_2_), depending on substrate availability and reaction conditions.^62^ Under the strongly basic aqueous conditions employed in HALT, however, difluorocarbene is also known to undergo competing hydrolysis with water, yielding formate and fluoride ions, as reported by Fier and Hartwig.^62^ This pathway is consistent with the products observed in the present study. Therefore, CF_2_: then rapidly reacts with OH^–^ and water to form difluoromethane alcohol (HCF_2_-OH), which is highly unstable and undergoes elimination to release HF, resulting in formyl fluoride (F-CHO) formation. Similar formation of F-CHO has been observed in the pyrolysis of PFOS in the presence of water.^63^ The F-CHO is subsequently attacked by OH^–^, leading to the formation of the observed organic intermediate formate, at which point defluorination is complete. Formate then reacts with OH^–^ to produce CO_3_^2–^ and H_2_ (confirmatory experiments described in Text S6 and Figure S6), in agreement with previous reports on formate hydrothermal reactions.^64,65^ The yield of H_2_ was consistent with the reaction stoichiometry depicted in Figure 1A: approximately 0.95 moles of H_2_ per mole of degraded TFMS. The net result of these reaction steps is the complete mineralization of TFMS to fluoride, sulfate, carbonate, and hydrogen. Similar degradation products were observed during HALT of trifluoroacetate (TFA), which occurs at much lower reaction temperatures (e.g., 200 °C),^59^ consistent with formation of a common CF_3_^–^ reaction intermediate (Figure S7). DFT calculations show that thermal C–C bond cleavage at the carboxylic acid head group in TFA only requires 15.6 kcal/mol, significantly lower than the calculated barrier for OH^–^ attack at the sulfonate head group of TFMS (27.8 kcal/mol), and consistent with the observed degradation of TFA at lower reaction temperatures.

Based on experimental and computational evidence, nucleophilic substitution and base-promoted hydrolysis reactions are the primary degradation and defluorination mechanisms during the HALT of TFMS/TFA. The initial desulfonation of TFMS and base-independent decarboxylation of TFA have the highest energy barriers along the compounds’ proposed pathways, making them the rate-determining steps (RDS). In these steps, the nucleophile (OH^–^) and reaction temperature are the primary driving factors. Previous studies have reported that CF_3_H exhibits significantly different reactivity with and without alkali during thermal treatment.^66^ Degradation of CF_3_H without alkali requires temperatures exceeding 600 °C, whereas required reaction temperatures drop to around 150 °C with alkali addition.^59,66^ This aligns with the absence of stable fluorinated intermediates during HALT of TFMS in this study (350 °C, 1 M NaOH), as confirmed by^19^F-NMR and LC-Orbitrap-MS nontargeted analysis of post-HALT aqueous samples, as well as GC-TCD analysis of headspace gas samples (see Figure S3). The RDS during HALT of TFMS is also consistent with earlier work examining PFOS degradation, which showed reaction rates dependent on OH^–^ concentration.^27^

Finally, the initial nucleophilic substitution reaction step is supported by hydrothermal reactions of TFMS with alternative nucleophiles leading to measurable defluorination (see Text S7 for details). Although TFMS reaction with bisulfide (HS^–^: 14.4 ± 0.3% defluorination) and iodide (I^–^: 0.6 ± 0.6% defluorination) yielded less defluorination than observed for OH^–^ (65.7 ± 2.3% defluorination) under the same reaction conditions,^55^ the findings are consistent with a nucleophilic substitution reaction occurring under subcritical reaction conditions. Additional research is suggested to investigate intermediates formed during the hydrothermal degradation of TFMS with alternative nucleophile species.

### Extension of Mechanism to Longer-Chain PFSAs

Building on the proposed mechanism for the TFMS (C_1_), a tentative mechanism is extended to describe transformation of longer-chain PFSAs. Although we previously proposed a hydroxide-mediated degradation pathway for PFOS (C_8_ PFSA),^27^ the proposed initial nucleophilic attack by OH^–^ on the α-carbon atom is less likely, based on the same molecular orbital rationale applied to TFMS.^55^ This is further supported by the marked instability of perfluoroalcohols under thermal and basic conditions, which is consistent with their role as degradation products formed after OH^–^ attack at the α-carbon.^67,68^ By contrast, perfluoroalkenes, expected from pathways involving OH^–^ attack at the headgroup, were reported by Haszeldine et al.^69^ during the thermal degradation of perfluoroalkyl carboxylates. Activation barrier calculations for the initial nucleophilic attack step were performed using perfluorobutane sulfonate (PFBS; C_4_ PFSA; Figure 3A), confirming the favorability of OH^–^ attack on the sulfonate group of longer chain PFSAs like PFBS, releasing SO_4_^2–^ and a long-chain perfluoroalkyl carbanion species [F_3_C­(CF_2_)_n_CF_2_^–^]. Once formed, the carbanion is proposed to follow a repeated series of reaction steps leading to complete mineralization (Figure S8a) that are similar to those proposed by Trang *et al.* ^37^ for perfluoroalkyl carboxylates reacting in NaOH-amended polar aprotic solvent mixtures with water (e.g., dimethylsulfoxide + 2.67 M NaOH, ≥80 °C).

**Figure 3:**
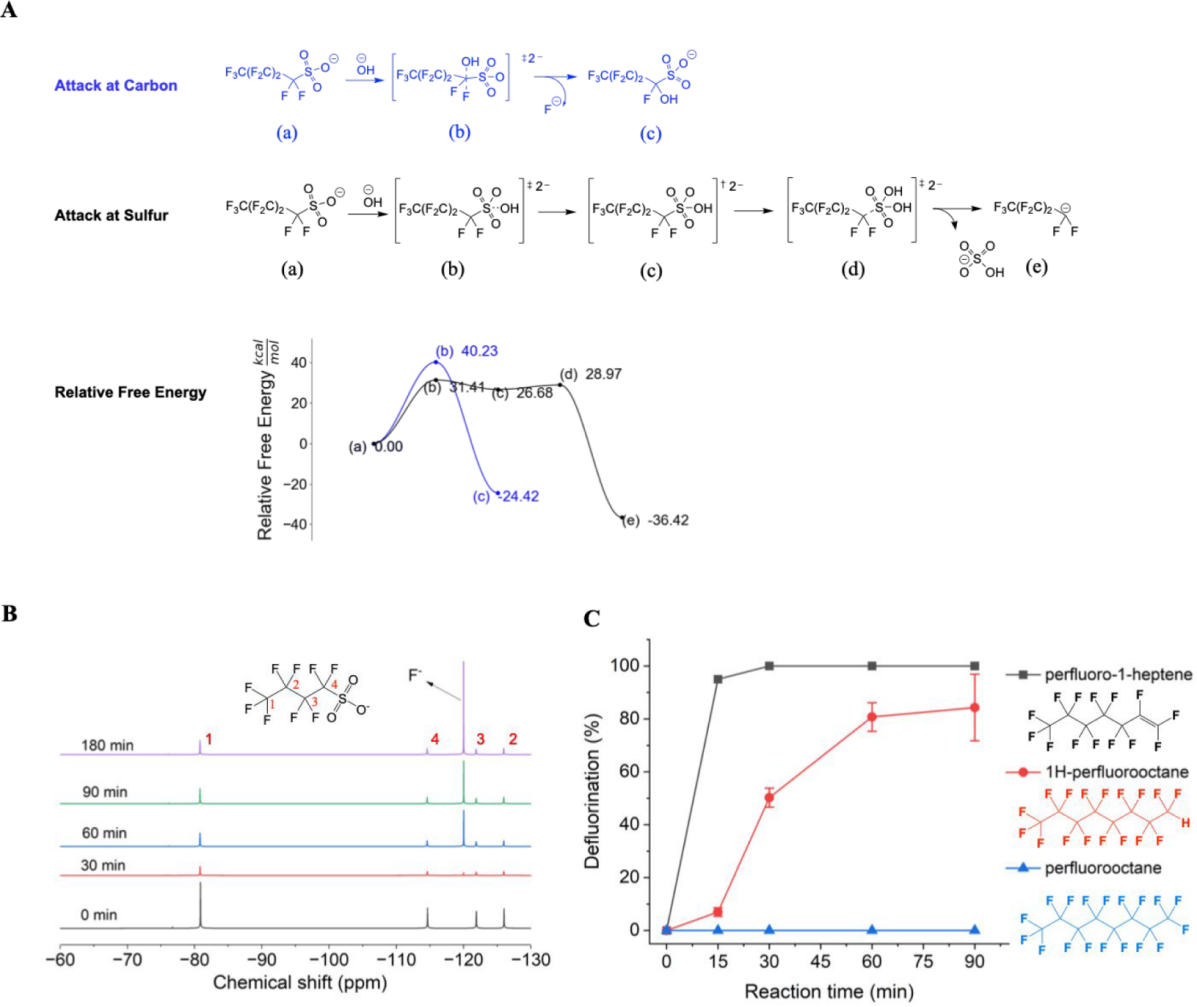
(A) Comparison of the energy barriers for OH^–^ attack on the carbon atom versus the sulfur atom in PFBS. (B) ^19^F-NMR tracking of HALT of PFBS (0.1 M) at 350 °C with 1 M NaOH. (C) Defluorination of perfluoro-1-heptene, 1H-perfluorooctane, and perfluorooctane during HALT (10 μL pure standard chemicals, 6 mL 1 M NaOH, and 350 °C).

Briefly, the perfluoroalkyl carbanion rapidly loses a fluoride ion, producing the perfluoroalkene CF_3_CF_2_CF=CF_2_, which then reacts with OH^–^ via nucleophilic substitution to form the intermediate CF_3_CF_2_CF=CFOH. This enol can eliminate HF, resulting in an α,β-unsaturated acyl fluoride (CF_3_CF=CF–CF=O). The remaining pathway proceeds through a series of addition and elimination reactions to achieve defluorination (Figure S8a). While TFA was reported as a final product of PFOA degradation in Trang’s study, TFA was not observed in HALT reactions because it is rapidly mineralized.^59^ As these fluorinated intermediates are highly reactive, confirming the mechanism through directly experimental detection of intermediates is challenging. As shown in Figure 3B, the ^19^F NMR spectra of a time series of aqueous samples from the HALT of PFBS over time show no new fluorine signals other than those from the parent compound and the terminal fluoride ion product. Although direct experimental evidence is not yet available (and may not be feasible, given the high reactivity of the proposed intermediates under hydrothermal conditions), DFT calculations for the HALT system initiated from a longer-chain perfluorinated carbanion show a trend consistent with Trang’s results (Figure S8b), further supporting the proposed base-promoted decomposition mechanism.

While validation of proposed mechanism by detecting fluorinated intermediates after HALT is not feasible, we instead experimentally examined the reactivity of one of the key proposed reaction intermediates. Reaction of perfluoro-1-heptene [CF_3_(CF_2_)_4_CF=CF_2_], an expected product following desulfonation of a long-chain PFSA (PFOS), was studied under HALT conditions. The reason for using perfluoro-1-heptene instead of perfluorobutene (the intermediate following desulfonation of PFBS) was its commercial availability at the time of the experiments. In addition, two other potential intermediates, 1H-perfluorooctane [CF_3_(CF_2_)_6_CF_2_H] and perfluorooctane [CF_3_(CF_2_)_6_CF_3_], previously reported for other thermal-based treatment technologies^70,71^ were obtained for study. All three compounds were subjected to HALT under the same conditions (Figure 3C). While perfluoro-1-heptene and 1H-perfluorooctane exhibited nearly complete defluorination when exposed to HALT conditions (350 °C, 1 M NaOH, and 90 min), no fluoride ion generation was detected for reaction of the fully fluorinated perfluorooctane (Figure 3C). This suggests that OH^–^ is unlikely to attack any carbon in the terminal CF_3_ group or randomly along the perfluoroalkyl carbon chain during reactions. Perfluoro-1-heptene was subjected to milder HALT conditions (200 °C, 1 M NaOH, 60 min), resulting in nearly 40% defluorination, whereas the parent PFSA (i.e., PFOS) was unreactive under these conditions. Thus, the much greater reactivity of perfluoro-1-heptene compared to PFOS is consistent with the fact that the former is not observed when subjecting the latter to HALT reaction conditions. Under strongly alkaline conditions (1 M NaOH), 1H-perfluorooctane [CF_3_(CF_2_)_6_CF_2_H] is likely to dissociate the weakly acidic proton to form the perfluorocarbanion [CF_3_(CF_2_)_6_CF_2_^–^].^37^ This perfluorocarbanion can then eliminate fluoride to generate perfluoro-1-octene [CF_3_(CF_2_)_5_CF=CF_2_]. Once formed, perfluoro-1-octene is expected to follow the defluorination pathway of long-chain PFSAs. This proposed mechanism is also consistent with the degradation pathway of 1H-perfluoroheptane in overheated water in the presence of dimethyl sulfoxide (DMSO) at mild temperatures (80–120 °C), as reported by Trang et al.^37^

Overall, OH^–^ plays a key nucleophilic role in attacking both TFMS and longer-chain PFSAs to form the initial perfluorocarbanion intermediates under hydrothermal conditions. The subsequent intermediates, including difluorocarbene and perfluoroalkenes, are highly reactive in near-critical water and undergo a series of base-promoted reactions that lead to complete defluorination without lag. Further research using more readily available intermediate compounds is needed to validate the individual steps in the proposed reaction pathways. We also acknowledge that additional side pathways may exist, particularly for long-chain PFAS such as PFOA, as suggested by Trang et al.^37^ However, proposing additional pathways based solely on computational predictions, without experimental evidence, would provide limited mechanistic value. Ongoing work is focused on comprehensively identifying additional transformation pathways for long-chain PFSAs and other polyfluorinated compounds, including fluorotelomers and ether-containing PFAS such as GenX.

### Factors Affecting PFSA Degradation Kinetics

Although the degradation kinetics of certain PFSAs (C_3_–C_8_) in real-world PFAS wastes (e.g., AFFF-impacted groundwater) and single PFOS solutions have been reported on previously,^25,27^ an in-depth investigation into the degradation kinetics of PFSAs was conducted here, extending to ultrashort chain structures (e.g., C_1_ TFMS and C_2_ PFEtS) in the clean matrix. In addition, influencing factors were examined to further support the proposed mechanism.

Five PFSAs with carbon chain lengths ranging from C_1_ to C_8_ were selected to study degradation kinetics (Figure 4A). The concentration of OH^–^ was set at 1 M, while the initial concentration of each PFSA was approximately 0.01–0.03 mM, containing up to a total fluorine content of up to 1.22 mM (further details in Text S8). This ensured that OH^–^ consumption as a nucleophile would be negligible according to Figure 1. At these conditions, the degradation kinetics of the five PFSAs (Figure 4A) display only a small chain-length dependence (*k*_obs_ values vary by a factor of 4), with shorter-chain PFSAs generally showing more recalcitrance than longer-chain analogues. Only small differences were observed across carbon chain lengths ranging from C_1_ to C_6_. In contrast to the orders-of-magnitude difference in reactivity across carbon chain lengths observed in other destructive treatment technologies, including UV/Sulfite,^40^ PFSAs demonstrated relatively uniform reactivity during HALT, further supporting its effectiveness and applicability for real-world remediation. In addition, the minimal chain-length dependence observed for PFSAs is consistent with the proposed mechanism, where the RDS involves the initial nucleophilic substitution and release of the sulfonate headgroup. DFT results indicated the energy barriers for PFSAs with different carbon-chain lengths were close. For example, the calculated barriers for reaction of TFMS and PFBS were 27.8 kcal/mol and 31.4 kcal/mol, respectively. The degradation of PFSAs appeared to follow a first-order kinetic model, with the measured apparent rate constants, *k*_obs_, shown in Table S2. This finding is consistent with our previous report.^25,27^ The slightly poor fitting of PFOS is likely due to adsorption of PFOS to sample-contact surfaces during handling and storage, which has been reported to cause negative bias in measured concentrations.^72,73^ It is also worth noting that factors such as solvent expansion/compression, real-time temperature and pressure profiles, and continuous-flow systems should be further evaluated to establish a more realistic kinetic model for real-world remediation conditions. Such work is currently underway, and preliminary results suggest that incorporating these factors improves the prediction of reaction kinetics.

**Figure 4:**
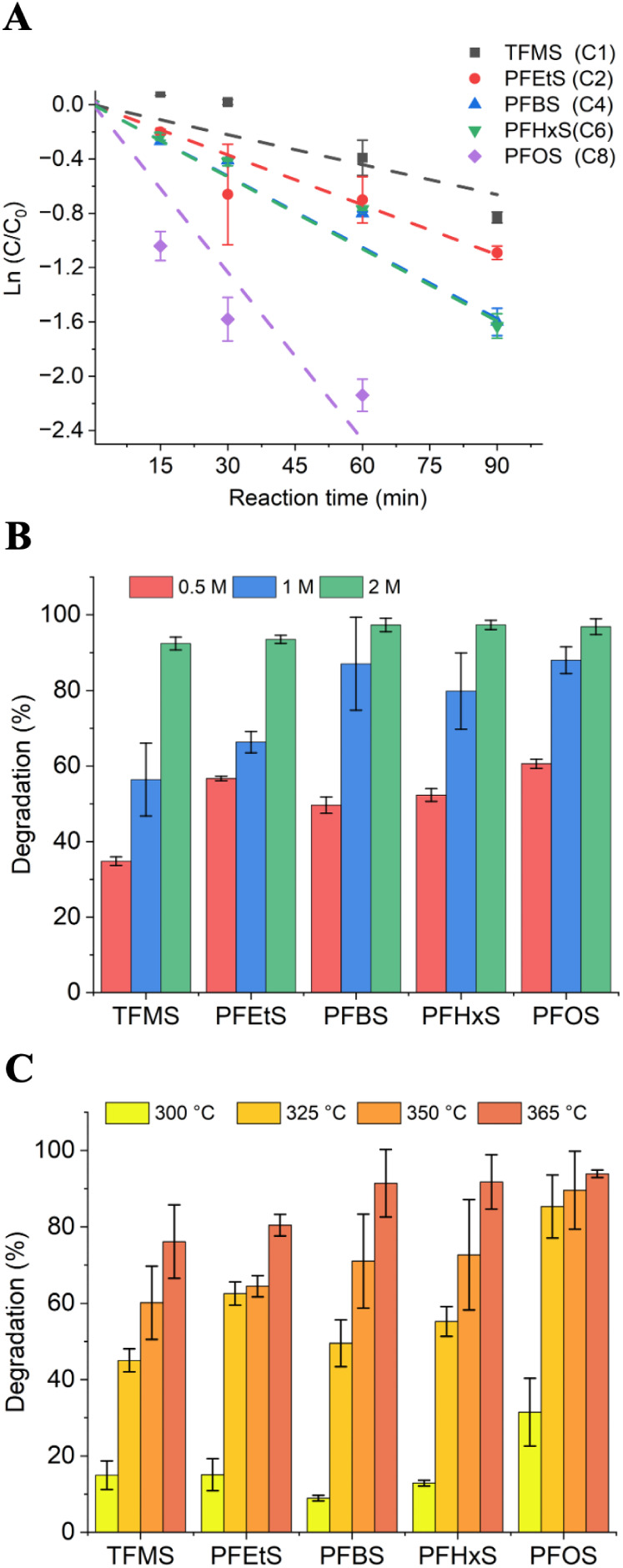
(A) Reaction kinetics of C1–C8 PFSAs during HALT under conditions of 350 °C and 1 M NaOH. Initial concentrations were 0.014 mM for TFMS, 0.033 mM for PFEtS, 0.014 mM for PFBS, 0.010 mM for PFHxS, and 0.019 mM for PFOS. (B) Effect of NaOH concentrations on the extent of degradation for individual PFSAs reacting at 350 °C for 90 min. No degradation was observed in the absence of NaOH. (C) Effect of reaction temperature on the extent of degradation of individual PFSAs reacting with 1 M NaOH for 60 min.

Consistent with previous observations in real-world environmental samples,^24,28^ higher temperatures and higher NaOH concentrations enhance the degradation of PFAS, particularly for PFSAs with fewer than eight carbons (Figure 4B and 4C). OH^–^ plays a dual role: it acts as a nucleophile to promote C–S cleavage through the initial nucleophilic substitution as outlined in the proposed mechanism, and is critical as a base, minimizing formation of volatile and thermally stable 1H-perfluoroalkane species and ensuring defluorination of the resulting perfluoroalkyl intermediates that form after the initial headgroup release. The proposed mechanism is also consistent with a prior report describing base-promoted hydrolytic decomposition of the fluorosulfate ion (SO_3_F^–^).^74^ Although fluorosulfate is not a PFAS, replacing the F substituent with a perfluoroalkyl group (e.g., CF_3_) yields a PFSA. This structural analogy provides a reasonable basis for the agreement between the two mechanisms.

### Environmental Implications

This study probed the underlying reaction mechanism leading to complete defluorination of PFAS via HALT, beginning with the C_1_ PFSA, TFMS. A complete mass balance for C, S, and F was achieved by qualitatively and quantitatively identifying all intermediates and final products, including carbonate, formate, sulfate, and fluoride. With the aid of DFT, the RDS was identified as the initial nucleophilic attack that cleaves the headgroup to form a perfluorinated carbanion. OH^–^ plays a dual role as both nucleophile and base reagent in achieving complete defluorination during HALT. The nucleophilic substitution and base-promoted mechanisms were then extended to longer-chain PFSAs. Due to the challenge of detecting stable fluorinated intermediates, the proposed mechanism for long-chain PFSAs relies more heavily on DFT calculations and prior literature. By evaluating the degradation kinetics of PFSAs with varying chain lengths, a slight chain-length dependence on reactivity was observed, with shorter-chain PFSAs generally being more recalcitrant. More aggressive conditions, including higher reaction temperatures and higher nucleophile concentrations, enhanced reaction kinetics of all PFSAs in a similar manner, further supporting a common underlying reaction mechanism.

It is noteworthy that the perfluoroalkene and 1H-perfluoroalkane intermediates exhibited much higher reactivity than PFSAs under the same conditions. This observation is consistent with their absence during treatment of highly recalcitrant PFSAs, which require elevated temperatures and high NaOH concentrations to degrade. Importantly, these species, although they may be considered products of incomplete destruction, are therefore not expected to persist as stable byproducts during HALT applications. At the same time, these byproducts, which are potent greenhouse gases, are likely to form during hydrothermal treatment of less-stable PFCAs in the absence of alkali. The risk of forming these species during thermal treatment processes (e.g., treatment of PFCA-contaminated organic wastes such as soils, biosolids, and foam concentrates) may be mitigated or eliminated through careful application of alkali amendments; however, further study is needed to confirm this effect.

Over the past decade, public attention to PFAS contamination and related regulatory policies worldwide has increased substantially. In response, remediation technologies have advanced rapidly, driven by investments from governments, industry, and funding agencies. As several PFAS destruction technologies, including HALT, supercritical water oxidation (SCWO), UV-based reduction, thermal incineration, electrochemical oxidation, and smoldering, advance toward pilot-scale, commercial, and full-scale implementation, understanding their underlying mechanisms and closing fluorine and carbon mass balances become increasingly important.^75,76^ Such understanding is needed to: (1) verify complete degradation and defluorination performance and minimize the risk of forming partially degraded intermediates; (2) track the fate of all reactants and identify whether additional disposal or treatment is required; and (3) provide mechanistic guidance for further optimization with lower operating costs.

Overall, the findings of this study provide an example of the mechanistic understanding needed for HALT technology, which is currently being employed for PFAS site remediation, and offer guidance for its further optimization and advancement. For example, this study is the first to close the carbon, sulfur, and fluorine mass balances for TFMS, an extremely recalcitrant PFAS, thereby providing greater confidence in the ability of HALT to achieve complete defluorination across a broad range of PFAS, particularly novel PFAS structures that are continually being identified through non-targeted analysis. In addition, the nucleophilic substitution mechanism suggested here indicates that other non-alkaline nucleophiles, such as NH_2_^–^, may be promising alternatives to OH^–^ for promoting defluorination during hydrothermal reactions. The search for alternative nucleophiles or solid base catalyst may help address the issue of residual NaOH in post-HALT solutions, which currently contributes substantially to the operating cost of HALT, according to the startup company which commercialized HALT, to date.

Lastly, the research approaches, tools, and analytical techniques employed in this study, including targeted and non-targeted analyses using LC-MS/MS, GC-MS, NMR, and IC, together with DFT calculations, provide a general framework for elucidating reaction mechanisms in other emerging PFAS destruction technologies where detailed mechanistic understanding is critical.

## Supplementary Material



## Data Availability

DFT-optimized geometries for the parent compound and proposed intermediates are available upon request.

## Notes

Additional information on chemical reagents and analytical methods is provided together with supporting figures and tables (PDF)
