# Supplementary material for: Mineralization of Trifluoromethanesulfonate via Subcritical Hydrothermal Reaction: Reaction Mechanism and Transformation Pathways
Source: Environ Sci Technol. 2026 Jun 25;60(26):18878–89. doi: 10.1021/acs.est.6c00018 (PMC13348019; doi:10.1021/acs.est.6c00018)
Supplement: Supplementary file 1 [file es6c00018_si_001.pdf]

Supporting Information for

Mineralization of Trifluoromethanesulfonate via Subcritical Hydrothermal Reaction: Reaction Mechanism and Transformation Pathways

Shilai Hao,[1,2,#] Benjamin Payton,[3,#] Ori Soker,[1] Anderson Ellis,[1] Sean Brooks,[1] Patrick Reardon,[4] Shubham Vyas,[3] Ilja Popovs,[5] Igor Novosselov,[6] Christopher P. Higgins,[1] Timothy J. Strathmann[1,]*

[1] Department of Civil and Environmental Engineering, Colorado School of Mines, Golden, Colorado 80401, USA

[2] School of Plant and Environmental Sciences, Virginia Tech, Blacksburg, Virginia, 24061, USA

[3] Department of Chemistry, Colorado School of Mines, Golden, Colorado 80401, USA

[4] NMR Facility, Oregon State University, Corvallis, Oregon 97331, USA

[5] Department of Nuclear Engineering, University of Tennessee, Knoxville, Tennessee 37996, USA

[6] Department of Mechanical Engineering, University of Washington, Seattle, Washington 98195, USA

[#]Shilai Hao and Benjamin Payton contributed equally to this work

*Corresponding author: Timothy J. Strathmann Email: strthmnn@mines.edu (T.J.S.)

This PDF file includes:

Section A: Supporting text S1 to S8
Section B: Figures S1 to S8
Section C: Table S1 to S2
Section D: SI References

Section A. Supporting information texts

Text S1. PFAS LC-Orbitrap-MS analysis

Sample preparation for PFAS analysis. Each sample before and after the hydrothermal reaction was diluted with LC-MS water to ensure that concentrations fall within the PFAS calibration range (5 – 5000 ng·L^{-1}). Following the recipe from a previous study,[1] A 400 µL solution containing 50% (v/v) sample, 50% (v/v) methanol, and 750 pg/mL internal standard (IS) was transferred to an HPLC vial for analysis.

PFAS targeted and nontargeted analysis. Samples were analyzed using an HPLC system coupled to an Orbitrap Exploris 240 high-resolution mass spectrometer (Thermo Scientific). Chromatographic separation was achieved on a Gemini C18 analytical column equipped with one C18 guard cartridge and two Zorbax DIOL guard columns for ESI− mode. The aqueous mobile phase consisted of two eluents: (A) 20 mM ammonium acetate (Fisher Scientific) in Optima® HPLC-grade water and (B) 100% Optima® HPLC-grade methanol. The flow rate was 0.60 mL/min with the following gradient: 0–0.5 min (90% A, 10% B), 0.5–8 min (50% A, 50% B), 8–13 min (1% A, 99% B), and 13–20 min (90% A, 10% B). Targeted and nontargeted analysis of PFASs was conducted using TraceFinderTM 5.1 and Compound Discoverer 3.4 to quantify targeted analytes and identify unknown PFASs based on MS/MS libraries and the NIST database. Additional methodological details are provided in the Supporting Information of a previous publication by Hao et al.[2]

Text S2. ^{13}C- and ^{19}F-NMR analysis

^{13}C- and ^{19}F-NMR analysis was conducted to track the fate of carbon and fluorine during hydrothermal reactions of PFSAs. Depending on instrument availability, samples were run on two NMRs including a JEOL ECA-500 spectrometer (500 MHz) and a Bruker Avance IIIHD 800 MHz spectrometer equipped with a 5 mm triple resonance cryogenic probe. Sodium trifluoroacetate or 2,2,2-Trifluoroethanol was added to samples as an internal standard (no overlap of fluorine signal between internal standards and individual PFSAs). D_2O was added to samples to lock the field of NMR. For ^{19}F-NMR conducted on a JEOL ECA-500 spectrometer, the spectral window was set from -200 to 200 ppm. The number of scans averaged was 128 and the acquisition time was 0.544 s. For ^{19}F-NMR conducted on a Bruker Avance IIIHD 800 MHz spectrometer, the spectral window was set from -239 to 19 ppm. The number of scans averaged was 128 and the acquisition time was 0.367 s. A 8.5 µs of the ^{19}F 90° pulse width and 15 s of relaxation delay were employed. For ^{13}C-NMR, the spectral window was set from -25 to 225 ppm. The number of scans averaged was 1000 and the acquisition time was 0.833 s. A 11.64 µs of the ^{13}C 90° pulse width and 2 s of relaxation delay were employed. The NMR raw data was processed using JEOL Delta v5.3.1 and MestReNova 14.3.

Text S3. IC analysis

The concentrations of sulfate (SO_4^{2-}) were measured by a Dionex ICS-90 ion chromatography (IC, Sunnyvale, CA). Specific separation method: Gradient, 1.0 mL min–1, 1−65 mM KOH, 30°C, 45 minutes. The concentrations of formate ($HCOO^-$) were measured by a Dionex ICS-5000 IC system. The IC system was equipped with a conductivity detector, a suppressor (AERS 4 mm), and a Dionex ICS6000 EG eluent generator using an EGC 500 KOH cartridge. An IonPac AS11-HC analytical column (4 × 250 mm) in line with an AG11-HC guard column (4 × 50 mm) was used for anion separation. Specific separation method: Gradient, 1.0 mL min–1, 1−65 mM KOH, 30°C, 45 minutes.

Text S4. TOC analysis and GC-TCD analysis

TOC Analysis. The TOC of samples before and after hydrothermal reactions were measured by a Shimadzu TOC-L analyzer (Columbia, MD) with the mode of TC (total carbon)/IC (inorganic carbon). A TFMS solution with 50 mg C /L was measured as a quality control experiment to test the ability of the TOC analyzer to mineralize PFASs. The capacity of TOC for measuring carbon content in PFOS was reported by Shimadzu.[3] We did observe the TOC analyzer gave lower TOC measurements with old catalyst (running > 1 year). The TOC values were calculated by subtracting IC from TC.

GC-TCD Analysis. Bulk gas-phase products (H_2 and CO_2) were analyzed using a gas chromatography with thermal conductivity detection (GC-TCD, ThermoFisher, Trace 1310). Individual gases (N_2, CO, and CO_2) with ultra-high purity grade purchased from General Air were used as standards compared against samples. Analyte separation was carried out on a Supelco Carboxen 1010 column (30 m length × 0.53 mm inter diameter × 30 μm thickness) with helium as carrier gas at a flow rate of 3 $mL{\cdot}min^{-1}$. Other settings included a split ratio of 2, injector temperature at 100°C, detector temperature set at 250°C, and filament temperature set at 300°C. The oven temperature was initially set at 35°C for 6 min, then ramped at a rate of 25°C/min to 220°C and held for 6.4 min.

Text S5. HALT experiment to test the reactivity of sulfite (SO_3^{2-})

A series of HALT experiments were conducted to test the reactivity of SO_3^{2-}. HALT conditions are 6 mL total volume, 50 mg/L SO_3^{2-}, 350 °C, 1M NaOH, and a reaction time of 60 min. Control experiments were performed under identical reaction conditions, except that the reaction was conducted at room temperature. A colorimetric method from a previous study[4] was used to measure the concentration of SO_3^{2-} in the post-HALT samples. The concentration of SO_4^{2-} was then measured by an IC with the method described in Text S3. The result shows that SO_3^{2-} was completely converted to SO_4^{2-} after HALT while SO_3^{2-} stayed unchanged under alkaline conditions at room temperature.

Text S6. HALT of formate to validate the degradation products and measure the degradation kinetics

A series of HALT experiments of formate were conducted to validate the degradation products and measure the degradation kinetics. HALT conditions are 6 mL total volume, 5000 mg/L formate, 350 °C, 1M NaOH, and reaction time ranging from 0-90 min. The concentrations of formate after HALT were measured by TOC using TC/IC method. The results from the TOC measurement were also confirmed by ion chromatography using the method shown in Text S4.

Text S7. Hydrothermal reaction of TFMS with alternative nucleophiles

Hydrothermal reactions of TFMS with alternative nucleophiles, including HS^- and I^-, were conducted to verify the nucleophilic substitution mechanism. Reaction conditions are 6 mL total volume, 50 mg/L TFMS, 350 °C, reaction time of 60 min, and 1 M nucleophiles including NaOH, NaHS, and KI. Following reaction, the concentration of F^- released was measured by FISE as described above.

Text S8. Hydrothermal reaction kinetics of PFSAs

Hydrothermal reactions of an approximately 5 mg/L PFSA mixture under different reaction conditions were conducted to investigate reaction influence factors and kinetics. The initial concentrations of each PFSA were as follows: TFMS (C1), 2.10 mg/L; PFEtS (C2), 6.61 mg/L; PFBS (C4), 4.45 mg/L; PFHxS (C6), 4.00 mg/L; and PFOS (C8), 9.32 mg/L. The variation in concentrations across PFSAs was attributed to uncertainty in sample preparation (e.g., weighing standards). The molar concentrations of the individual PFSAs are provided in the table below. Varying reaction conditions include reaction temperatures (250-350 °C), NaOH concentrations (0-1 M), and reaction time (0-90 min).

**PFAS name**	**Molecular weight (g/mol)**	**Mass concentration (mg/L)**	**Molar concentration (mM)**
TFMS	150	2.10	0.014
PFEtS	200	6.61	0.033
PFBS	300	4.45	0.014
PFHxS	400	4.00	0.010
PFOS	500	9.32	0.019

Section B. Supporting information figures

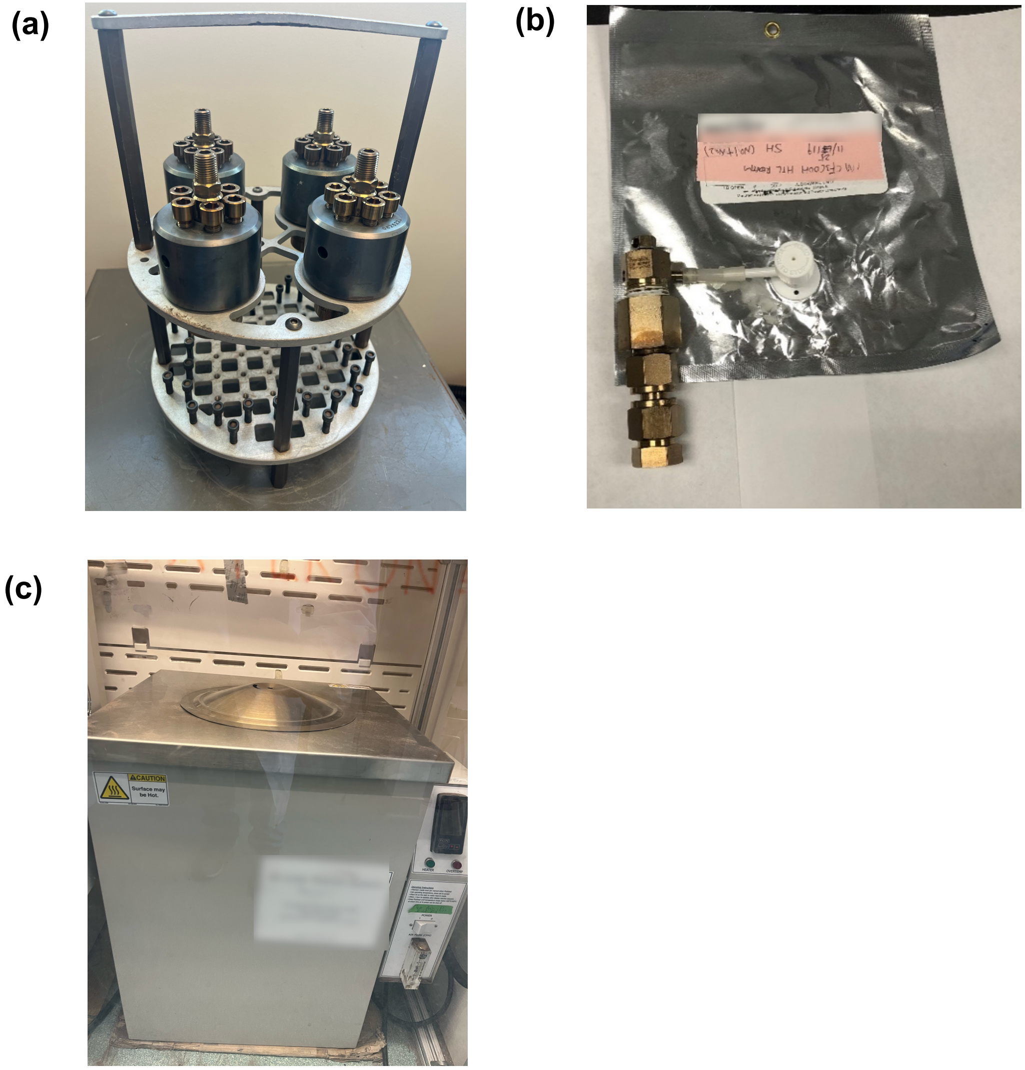

Figure S1. (a) Parr Reactors with a reactor stand, (b) mini-tube reactor vessel with a gas valve to collect headspace gas samples, and (c) temperature-controlled fluidized sand bath used for heating reactors.

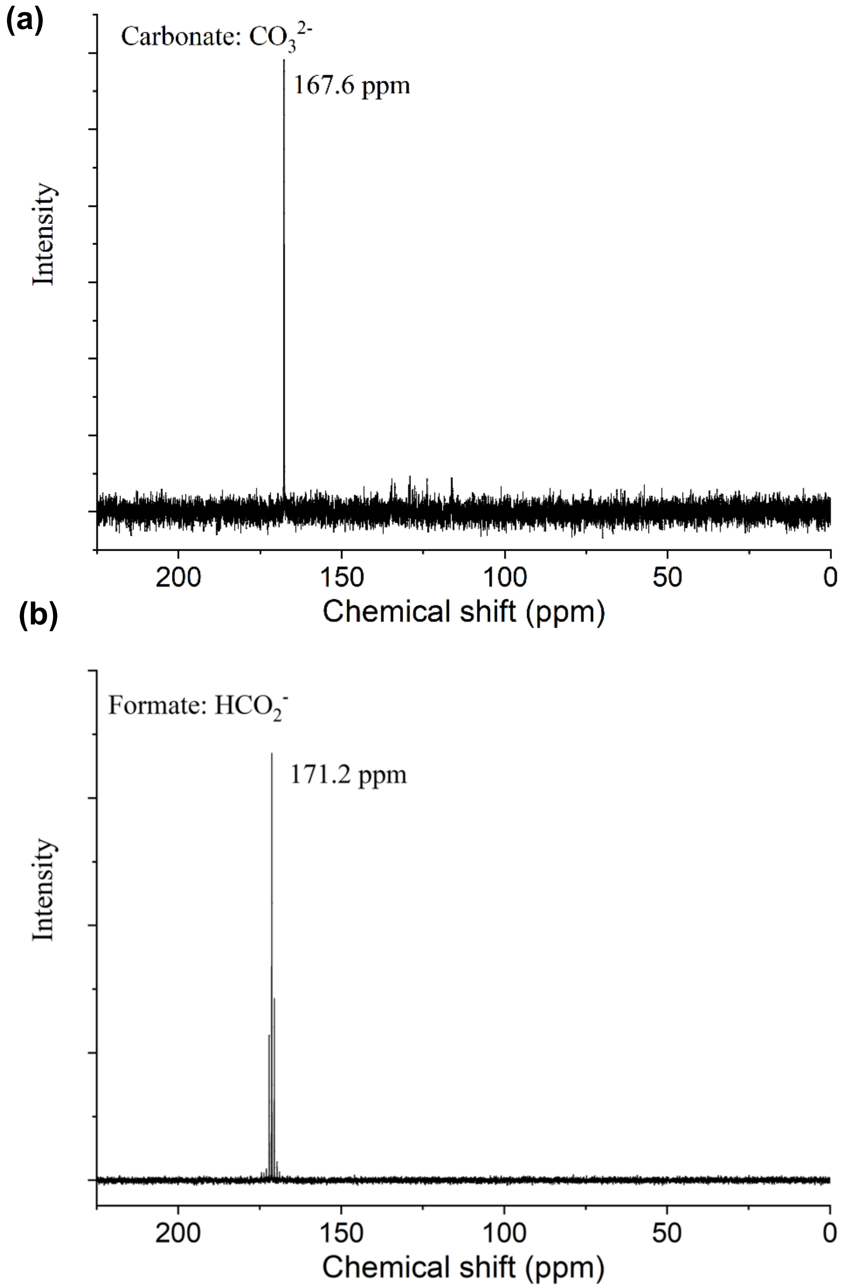

Figure S2. ^{13}C-NMR spectra of (a) 1 M Na_2CO_3 in water and (b) 1 M HCOONa in water.

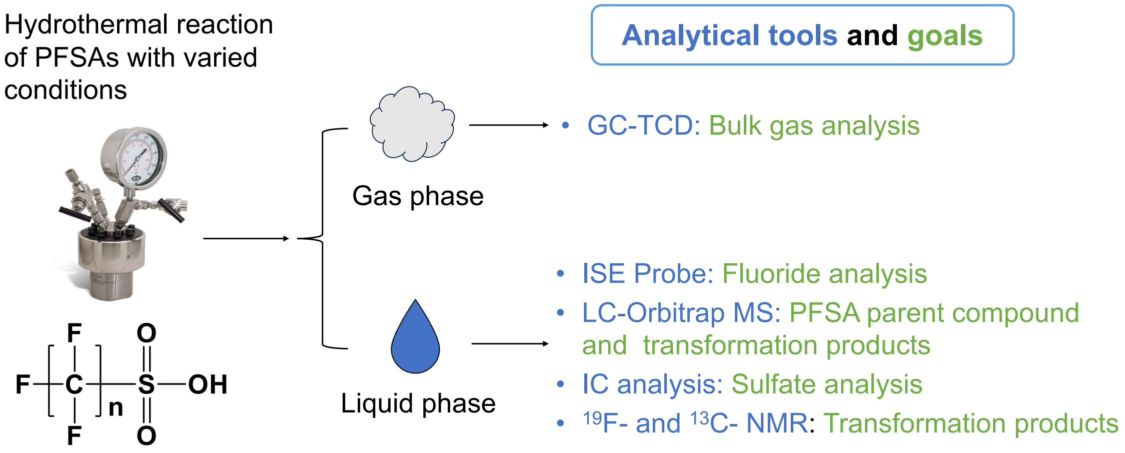

Figure S3. The schematic of analytical tools and goals for samples from the hydrothermal reaction of PFSAs.

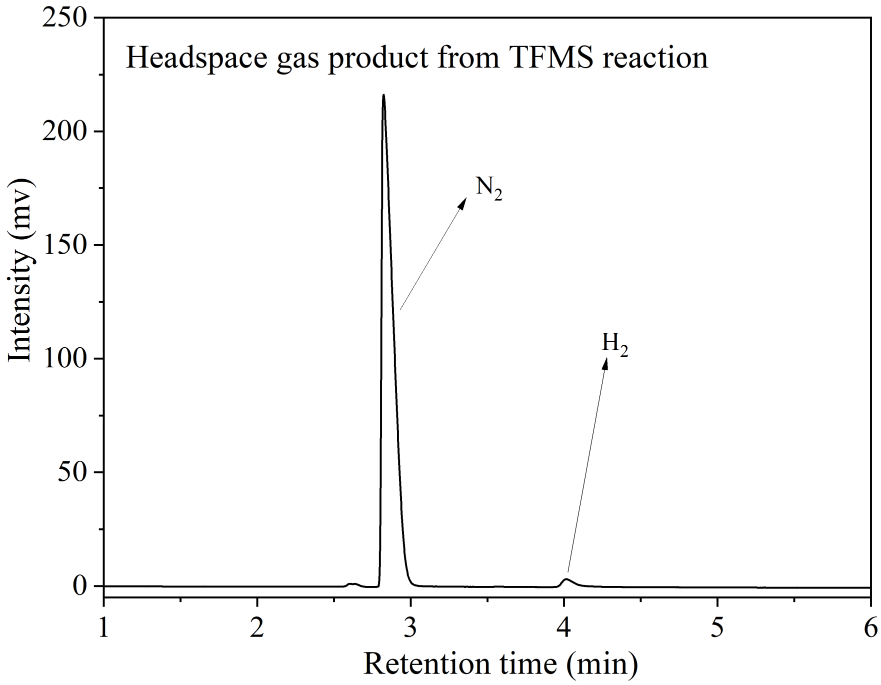

Figure S4. GC–TCD analysis of gas products in the reactor headspace from HALT of TFMS under the following conditions: 149 g/L (1 M) TFMS, 350 °C, 1 M NaOH, and a reaction time of 90 min. The reaction was conducted in the reactor equipped with a gas sampling valve, as shown in Figure S1B, and the loading volume of TFMS solution was 1 mL. As 65.7% of TFMS was degraded, 0.657 mmol TFMS was mineralized to produce H_2. According to the reaction stoichiometry shown in Figure 1, 0.657 mmol H_2 would be generated, corresponding to 14.7 mL of H_2 assuming ideal gas behavior. The N_2 peak originated from residual air remaining in the headspace because the reactor was not purged prior to the reaction.

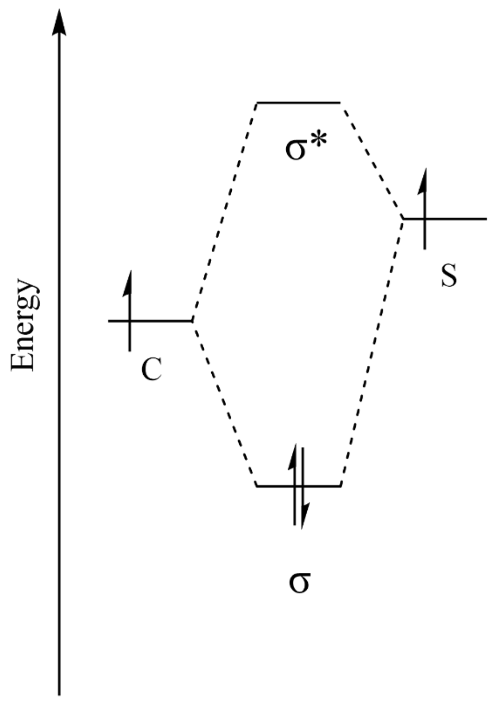

Figure S5. Simplified molecular orbital diagrams of the C–S bond, illustrating that the carbon-centered orbital is lowered in energy by the electron-withdrawing fluorine atoms, while the higher-energy sulfur valence shell contributes more strongly to the antibonding orbital. This electronic structure makes sulfur the preferred site for nucleophilic attack by hydroxide.

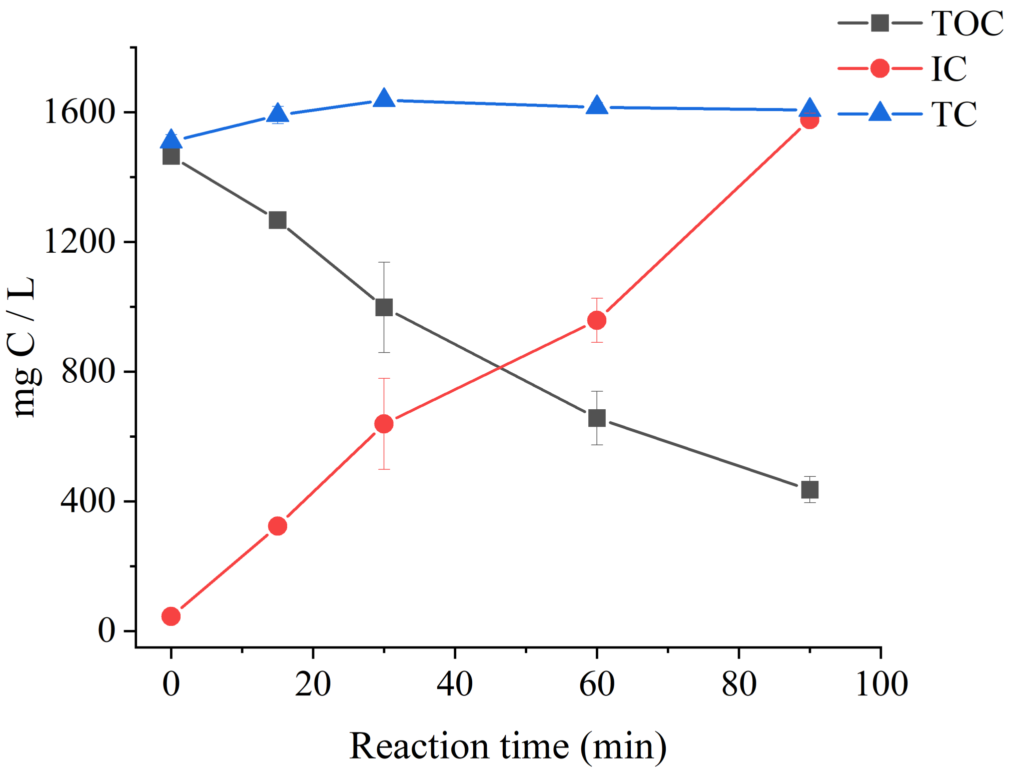

Figure S6. TOC tracking of HALT of 5000 mg/L formate (TC:1333 mg C/L) under reaction conditions: 350 °C, 1 M NaOH, and 0-90 min.

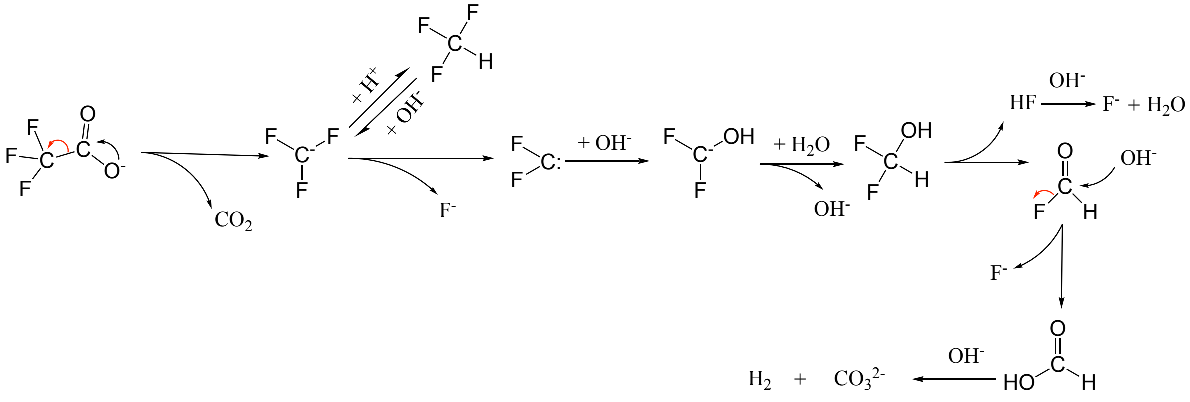

Figure S7. Proposed defluorination pathway of TFA during HALT.

(a)

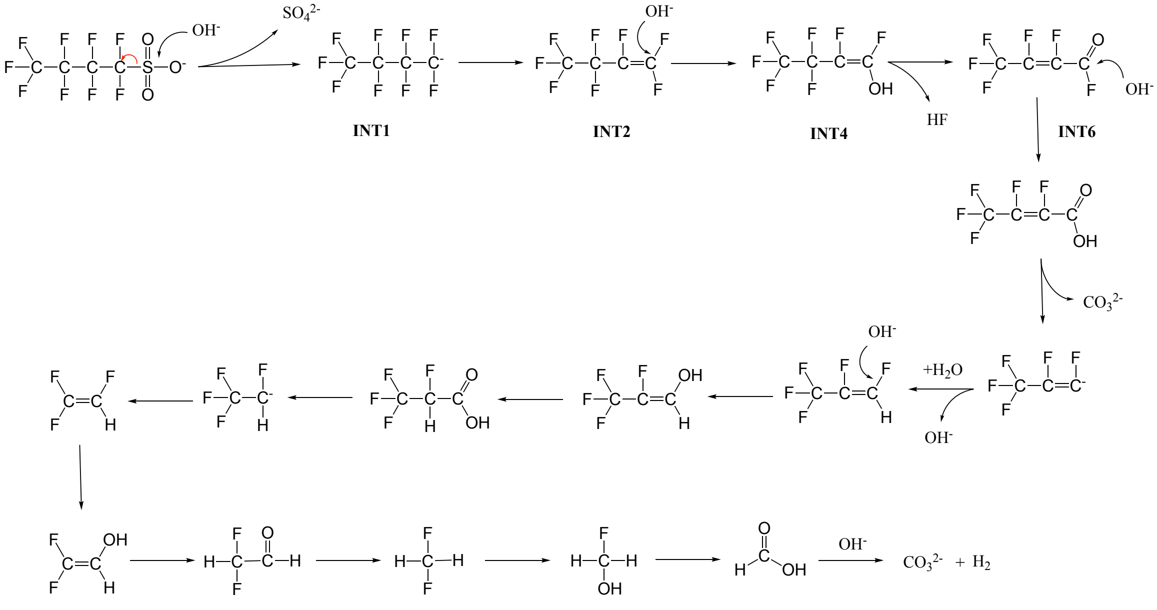

(b)

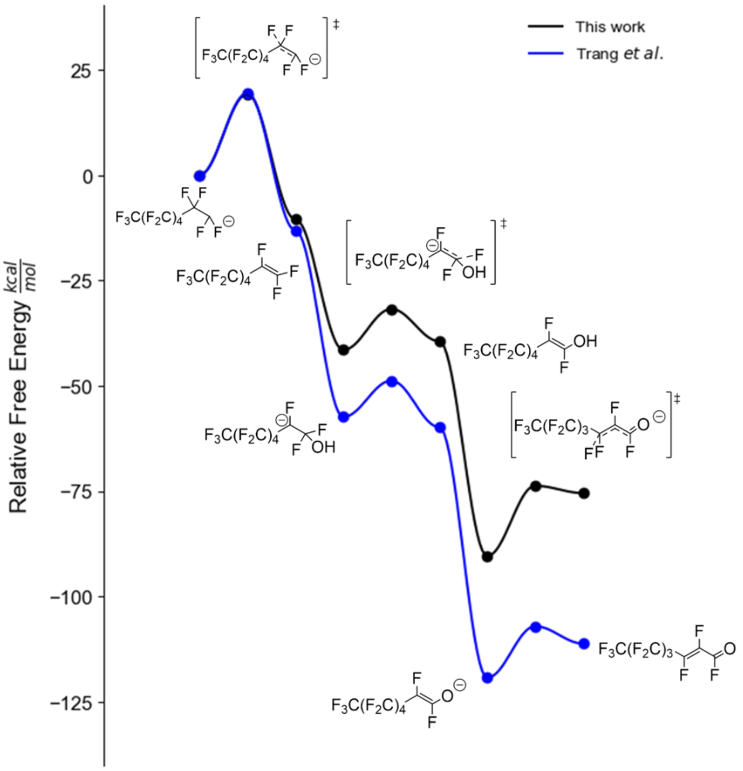

Figure S8. (a) Proposed HALT defluorination pathway for PFBS (C4). Intermediate numbers are assigned following Trang's study to facilitate comparison of DFT results; (b) Comparison of DFT-calculated energies for intermediates 1–6 from PFOA degradation along the pathway reported by Trang *et al.*[5], as obtained in this work (black line) and in Trang's original calculations (blue line).

Section C. Supporting information table

Table S1. Chemical reagents.

Reagent	Purity	CAS#	Supplier
Sodium trifluoromethane sulfonate (TFMS)	98%	2926-30-9	Sigma-Aldrich, USA
Perfluoroethane sulfonate (PFEtS)	98%	354-88-1	Synquest Laboratories Inc
Perfluorobutane sulfonate (PFBS)	97%	375-73-5	Sigma-Aldrich, USA
Potassium perfluorohexanesulfonate (PFHxS)	certified reference material,	3871-99-6	Sigma-Aldrich, USA
Potassium perfluorooctane sulfonate (PFOS)	>98%	2795-39-3	Sigma-Aldrich, USA
Perfluorooctanoic acid (PFOA)	Analytical standard	335-67-1	Sigma-Aldrich, USA
1H-Perfluorooctane	99%	335-65-9	Combi-Blocks
Perfluorooctane	98%	307-34-6	Sigma-Aldrich, USA
NaHS	>67%	207683-19-0	Fisher Scientific, USA
KI	>98%	7681-11-0	Fisher Scientific, USA
HCl	30 wt.% in water	7647-01-0	Sigma-Aldrich, USA
NaOH	97%	1310-73-2	Merck, Germany
NaF	99%	7681-49-4	Sigma-Aldrich, USA
D_2O	99.96 atom % D	7789-20-0	Sigma-Aldrich, USA
Ammonium acetate	Optima LC-MS grade	631-61-8	Fisher Scientific, USA
Ammonium hydroxide	Optima grade	1336-21-6	Fisher Scientific, USA
Methanol	Optima LC-MS grade	67-56-1	Fisher Scientific, USA
Isopropanol	Optima LC-MS grade	67-63-0	Fisher Scientific, USA
Trifluoroethanol	99.8%	75-89-8	Fisher Scientific, USA
Water (for LC-MS/MS)	Optima LC-MS grade	7732-18-5	Fisher Scientific, USA
Acetic acid	≥99%	64-19-7	Sigma-Aldrich, USA

Table S2. Measured apparent rate constants, k_{obs}, for individual PFSAs under 350 °C and 1 M NaOH conditions.

Perfluoroalkyl sulfonate	Abbreviation (#C)	k_{obs} (min^{-1})
Trifluoromethane sulfonic acid	TFMS (1)	0.0125 ± 0.0012
Pentafluoroethane sulfonic acid	PFEtS (2)	0.0114 ± 0.0010
Perfluorobutane sulfonic acid	PFBS (4)	0.0174 ± 0.0020
Perfluorohexane sulfonic acid	PFHxS (6)	0.0189 ± 0.0024
Perfluorooctane sulfonic acid	PFOS (8)	0.0404 ± 0.0050

Section D: SI References

(1) Nickerson, A.; Maizel, A. C.; Kulkarni, P. R.; Adamson, D. T.; Kornuc, J. J.; Higgins, C. P. Enhanced Extraction of AFFF-Associated PFASs from Source Zone Soils. *Cite This: Environ. Sci. Technol* 2020, *54*, 4962. https://doi.org/10.1021/acs.est.0c00792.

(2) Hao, S.; Reardon, P.; Choi, Y. J.; Zhang, C.; Sanchez, J.; Higgins, C.; Strathmann, T. Hydrothermal Alkaline Treatment (HALT) of Foam Fractionation Concentrate Derived from PFAS-Contaminated Groundwater. *Environ Sci Technol 57* (44), 17154–17165. https://doi.org/10.1021/acs.est.3c05140.

(3) *Measurement of TOC in Perfluorochemical (PFOS) Solution : SHIMADZU (Shimadzu Corporation)*. https://www.shimadzu.com/an/literature/toc/jph515005.html (accessed 2024-06-14).

(4) Tenorio, R.; Liu, J.; Xiao, X.; Maizel, A.; Higgins, C. P.; Schaefer, C. E.; Strathmann, T. J. Destruction of Per-and Polyfluoroalkyl Substances (PFASs) in Aqueous Film-Forming Foam (AFFF) with UV-Sulfite Photoreductive Treatment. *Environ. Sci. Technol* 2020, *54*, 6957–6967. https://doi.org/10.1021/acs.est.0c00961.

(5) Trang, B.; Li, Y.; Xue, X. S.; Ateia, M.; Houk, K. N.; Dichtel, W. R. Low-Temperature Mineralization of Perfluorocarboxylic Acids. *Science (1979)* 2022, *377* (6608), 839–845. https://doi.org/10.1126/SCIENCE.ABM8868/SUPPL_FILE/SCIENCE.ABM8868_SM.PDF.
